# A Method for Identifying the Spatial Range of Mining Disturbance Based on Contribution Quantification and Significance Test

**DOI:** 10.3390/ijerph19095176

**Published:** 2022-04-24

**Authors:** Chengye Zhang, Huiyu Zheng, Jun Li, Tingting Qin, Junting Guo, Menghao Du

**Affiliations:** 1 College of Geoscience and Surveying Engineering, China University of Mining and Technology—Beijing, Beijing 100083, China; czhang@cumtb.edu.cn (C.Z.); zhenghy@student.cumtb.edu.cn (H.Z.); qting@student.cumtb.edu.cn (T.Q.); menghaodu@student.cumtb.edu.cn (M.D.); 2 State Key Laboratory of Water Resource Protection and Utilization in Coal Mining, Beijing 102209, China; junting.guo.a@chnenergy.com.cn

**Keywords:** disturbance range, GWANN, fractional vegetation cover, mining area, environment

## Abstract

Identifying the spatial range of mining disturbance on vegetation is of significant importance for the plan of environmental rehabilitation in mining areas. This paper proposes a method to identify the spatial range of mining disturbance (SRMD). First, a non-linear and quantitative relationship between driving factors and fractional vegetation cover (FVC) was constructed by geographically weighted artificial neural network (GWANN). The driving factors include precipitation, temperature, topography, urban activities, and mining activities. Second, the contribution of mining activities (*W_mine_*) to FVC was quantified using the differential method. Third, the virtual contribution of mining activities (*V-W_mine_*) to FVC during the period without mining activity was calculated, which was taken as the noise in the contribution of mining activities. Finally, the SRMD in 2020 was identified by the significance test based on the *W_mine_* and noise. The results show that: (1) the mean RMSE and MRE for the 11 years of the GWANN in the whole study area are 0.0526 and 0.1029, which illustrates the successful construction of the relationship between driving factors and FVC; (2) the noise in the contribution of mining activities obeys normal distribution, and the critical value is 0.085 for the significance test; (3) most of the SRMD are inside the 3 km buffer with an average disturbance distance of 2.25 km for the whole SRMD, and significant directional heterogeneity is possessed by the SRMD. In conclusion, the usability of the proposed method for identifying SRMD has been demonstrated, with the advantages of elimination of coupling impact, spatial continuity, and threshold stability. This study can serve as an early environmental warning by identifying SRMD and also provide scientific data for developing plans of environmental rehabilitation in mining areas.

## 1. Introduction

Mining usually has a negative disturbance on the surrounding environment at different degrees, which leads to a variety of environmental issues (e.g., vegetation reduction, land degradation). Sustainable development and public health require essential environmental remediation in mining areas. It is of vital importance for the plan of environmental remediation to exactly identify the spatial range of mining disturbance (SRMD).

In the past decades, the impact of mining disturbance on the environment has been investigated by scholars. First, the impact of mining on vegetation was analyzed by satellite images and field surveys [1,2,3]. For example, the forest loss due to mining activities was assessed by combining high-resolution satellite images [1]. Second, several investigations improved the previous methods for assessing the impact of mining disturbances or proposed new indicators to evaluate the environmental quality in mining areas [4,5,6]. For instance, the remote sensing ecological index (RSEI) was improved to form a moving window remote sensing ecological index (MW-RSEI) to evaluate the environment of mining areas [6]. An indicator was constructed to evaluate the impacts on the environment under different mining intensities [5]. Third, a part of investigations reconstructed the disturbance history of vegetation in mining areas. For example, the duration of the mining disturbance was analyzed using 28 years of Landsat images [7]. Although the impacts of mining disturbances have been extensively investigated, these investigations have not involved how to identify the SRMD.

In recent years, some scholars have started to explore the identification of SRMD, and these publications can be divided into two categories. One category is to fit the trend of vegetation indices using empirical modeling methods, and a threshold has to be determined artificially at the stable asymptote to differentiate the area disturbed by mining activities from that without disturbance. For example, the trend of temperature vegetation dryness index (TVDI) was fitted using a linear model [8]. The trend of NDVI based on the coefficient of variation (CV) [9,10,11] and the LandTrendr algorithm [12] was fitted. The other category is to identify the SRMD based on a comparative area. For example, the comparison of the trends of vegetation indices at different distances was conducted to identify the SRMD based on the Seasonal-Trend decomposition procedure based on Loess (STL) and principal component analysis [13].

However, existing studies still have the following shortcomings. First, the identification of SRMD was based on direct observations of vegetation indices, which attributed the changes in vegetation exclusively to mining activities [9,10,11,12,13,14]. Nevertheless, the change of vegetation in the real situation is the result of coupling multiple factors (e.g., temperature, precipitation, topography, mining activities, etc.). Existing studies have not separated the mining factor from other factors, which usually resulted in improperly taking the areas impacted by other factors such as SRMD. In some areas, changes in vegetation cover may be caused by topography or climate but not by mining. For example, the decrease in vegetation cover was caused by the increase in topography but was incorrectly attributed to mining. Second, the trend of the vegetation index is fitted in sample areas selected from different directions or in buffers generated at different distances. The value of the stable trend is determined as the threshold for extracting the SRMD [8,9,10,11,12]. However, errors have been brought by the artificial determination of thresholds. Third, the spatial discontinuity between the sample areas creates blind areas in the study (i.e., some areas could not be covered for the disturbance analysis) [10,13]. Hence, the existing methods are still limited, and it is necessary to propose new methods to make up for the above deficiencies and provide more optional methods for the identification of SRMD.

In this paper, a method is proposed for identifying the SRMD by quantifying the contribution of mining activities and separating out the mining factor from other factors in the Shengli Mine and Xi-2 Mine in Inner Mongolia, China. First, a geographically weighted artificial neural network (GWANN) is used to construct the relationship between fractional vegetation cover (FVC) and driving factors (i.e., precipitation, temperature, topography, urban activities, and mining activities). Second, the contribution of mining activities to FVC is quantified using the differential method. Third, the noise in the contribution of mining activities is quantified in the period without mining activity (1992–2003), and the virtual contribution is taken as this noise. Finally, the SRMD in 2020 is identified using a significance test and the contribution of mining activities in 2020. To the best of our knowledge, this paper is the first to identify the SRMD based on quantifying the contribution of the mining factor, which avoids the coupling impacts from multiple factors. In addition, the artificial determination of a threshold is also avoided by using a significance test, and the SRMD identified by the proposed method has the advantage of spatial continuity.

## 2. Study Area and Datasets

### 2.1. Study Area

**Figure 1:**
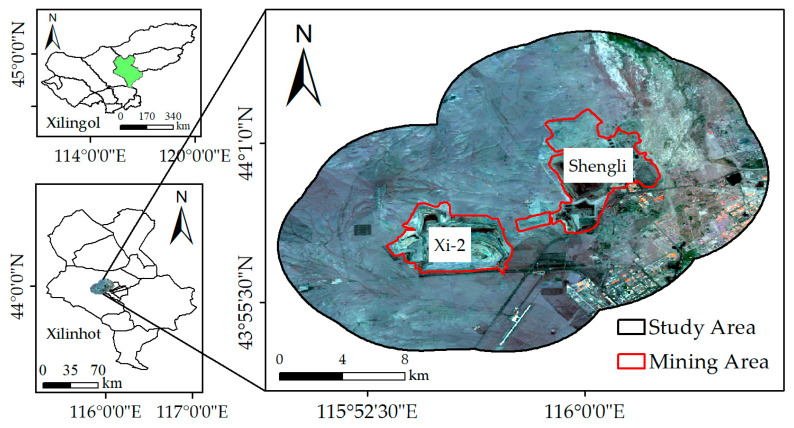
Location of the study area.

The study area (115°48′ E~116°06′ E, 43°30′ N~44°50′ N) is in Xilinhot, Inner Mongolia, China, covering the Shengli Mine and Xi-2 Mine, as shown in Figure 1. It belongs to the mid-temperate semi-arid continental monsoon climate. The land-use types in the study area mainly include grassland, woodland, bare land, cultivated land, mining areas, and impervious surface. The grassland types include meadow grassland, typical grassland, and dune sand grassland. With the temporal evolution, the area of mining areas and impervious surface has been increasing, and their direct occupation of the grasslands has resulted in a decrease in grassland area. In addition, there are large areas of well-grown woodland to the northeast of the study area.

In the past 20 years, the annual average temperature has been 3.25 °C, and the annual average precipitation has been 275.00 mm, mainly concentrated from June to August. The elevation of the study area ranges from 939 m to 1319 m, and the average elevation is 1129 m. In addition, the topography of the study area shows a high west and low east.

As the largest lignite field in China, the coal resources of Shengli Mine and Xi-2 Mine have enormous potential value [15], and the mining activities in the study area started in 2004. In addition to the direct destruction inside the mining area, mining activities also have an indirect impact on the vegetation around the mining area.

### 2.2. Datasets

Landsat images, precipitation data, temperature data, topography data, urban activities data, and mining activities data are utilized in this study.

Landsat images from 1992 to 2020, including Landsat 5 Thematic Mapper (TM), Landsat 7 Enhanced Thematic Mapper Plus (ETM+), and Landsat 8 Operational Land Imager (OLI), were obtained for this study. The surface reflectance (SR) dataset with the spatial resolution of 30 m was used in this study, which was produced by the National Aeronautics and Space Administration (NASA) and United States Geological Survey (USGS) [16] and was available on Google Earth Engine (GEE) [17]. In particular, the SR dataset is the surface reflectance data after essential pre-processing (i.e., radiometric calibration and atmospheric correction). The years 1993 and 1994 were excluded due to the poor quality of the images. The band information of Landsat images is shown in Table 1.

The precipitation and temperature datasets were obtained from the website of China Meteorological Data (http://data.cma.cn (accessed on 2 February 2021)). The datasets include monthly accumulated precipitation (unit: mm) and monthly mean temperature (unit: °C) for Xilinhot station (station number 54,102) from 1992 to 2003 (except 1993 and 1994) and 2020.

**Figure 2:**
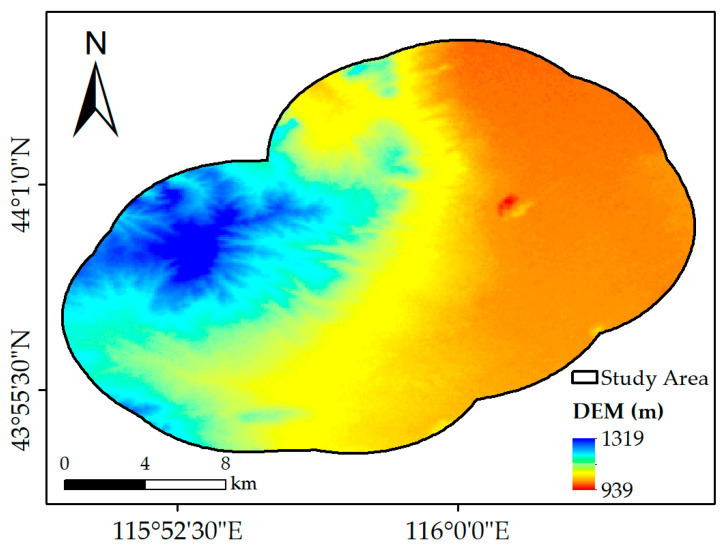
The spatial distribution of DEM.

The data for the topography were obtained from the digital elevation model (DEM). The DEM used in this study was the Advanced Spaceborne Thermal Emission and Reflection Radiometer Global DEM (ASTER GDEM) dataset provided by NASA [18,19]. The coverage of the ASTER GDEM extends from 83° N to 83° S, covering almost all the land on the Earth, which has been widely used in the analysis of vegetation change [20]. For this dataset, the horizontal accuracy is 30 m, while the vertical accuracy is 20 m [21]. The DEM used in this study is shown in Figure 2.

The data on urban activities and mining activities include the population and the coal production in Xilinhot. The specific description of the data is shown in Table 2.

## 3. Methods

### 3.1. Inversion of FVC

*FVC* refers to the ratio of the area of vegetation (including leaves, stems, branches, etc.) vertically projected on the ground in each pixel to the total area of the pixel [22,23]. The value of *FVC* can objectively and accurately reflect the spatial distribution of vegetation in the study area. The steps for obtaining *FVC* were described as follows.

Landsat images from July 1 to September 30 each year were used to calculate NDVI (Equation (1)) when the vegetation was growing most vigorously in the study area. The maximum *NDVI* at each pixel from July to September for each year was calculated using the maximum algorithm in GEE.

(1) $$\mathrm{NDVI}=\frac{\rho_{\mathrm{NIR}}-\rho_{\mathrm{Red}}}{\rho_{\mathrm{NIR}}{+\rho }_{\mathrm{Red}}}$$

where *ρ_NIR_* is the surface reflectance in the near-infrared band and *ρ_Red_* is the surface reflectance in the red band.

The correlations of the *NDVI* between different sensors (TM and ETM+, ETM+ and OLI) were established using the least-squares fitting method proposed by [24] based on overlapped images. ETM+ was used as an intermediate sensor to establish the correlation of *NDVI* between TM and OLI, as there was no overlapped operating time between Landsat 5 and Landsat 8. Then, the NDVI of both TM and ETM+ were corrected to OLI based on the established correlations to eliminate the systematic bias caused by different sensors. The above method has been widely used in many publications [25,26,27].

In this study, *FVC* was calculated by NDVI using the pixel dichotomy model, as shown in Equation (2). The pixel dichotomy model is one of the most widely used methods to calculate *FVC* [28,29].

(2) $$\mathrm{FVC}=\frac{\mathrm{NDVI}-{\mathrm{NDVI}}_{\min}}{{\mathrm{NDVI}}_{\max}-{\mathrm{NDVI}}_{\min}}$$

where *NDVI* is the *NDVI* of the pixel, *NDVI_min_* is the *NDVI* of pure soil, and *NDVI_max_* is the NDVI of pure vegetation.

**Figure 3:**
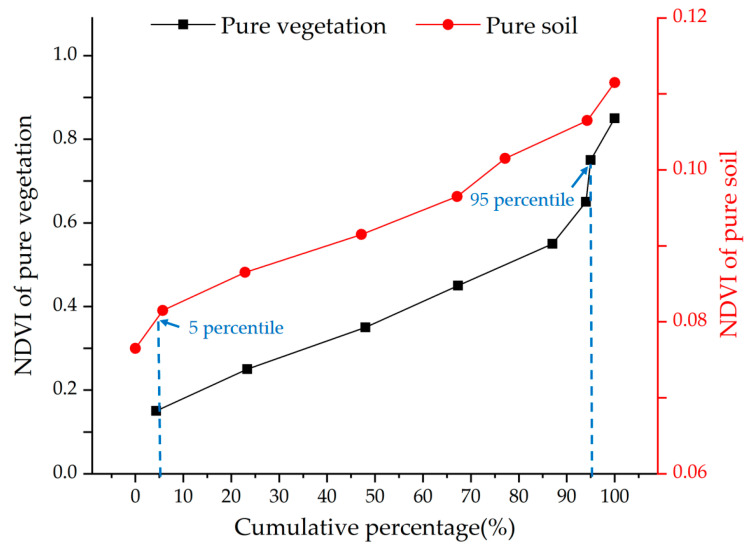
The cumulative percentage of NDVI in pure vegetation pixels and pure soil pixels.

**Figure 4:**
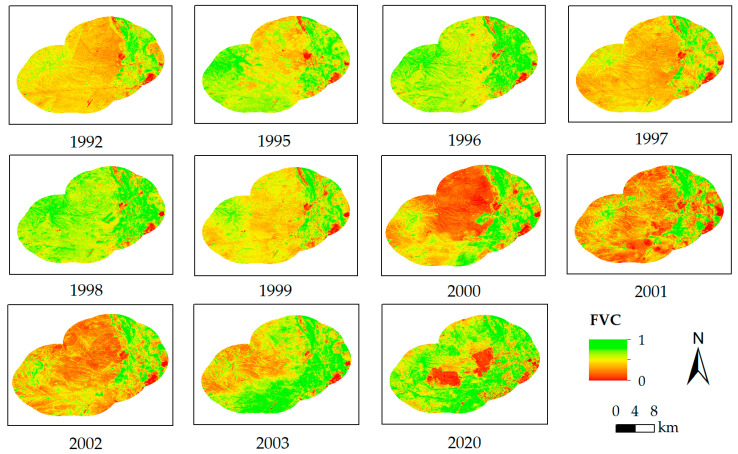
The spatial distribution of FVC from 1992 to 2003 (except 1993 and 1994) and 2020.

*NDVI_min_* and *NDVI_max_* were determined as follows. The pixels of completely bare soil were selected, and the NDVI was calculated from 1992 to 2003 (except 1993 and 1994) and 2020. To avoid extreme anomalous values due to noise, these NDVI were sorted in ascending order, and the pixel value corresponding to a cumulative percentage of 5 was used as the *NDVI_min_*. Then, the pixels of completely vegetation cover were selected, and the NDVI was calculated from 1992 to 2003 (except 1993 and 1994) and 2020. These NDVI were sorted in ascending order, and the pixel value corresponding to a cumulative percentage of 95 was used as the *NDVI_max_*. In this study, the *NDVI_min_* was determined as 0.08, while the *NDVI_max_* was 0.7, as shown in Figure 3. The FVC is shown in Figure 4.

### 3.2. Spatialization of Driving Factors

**Figure 5:**
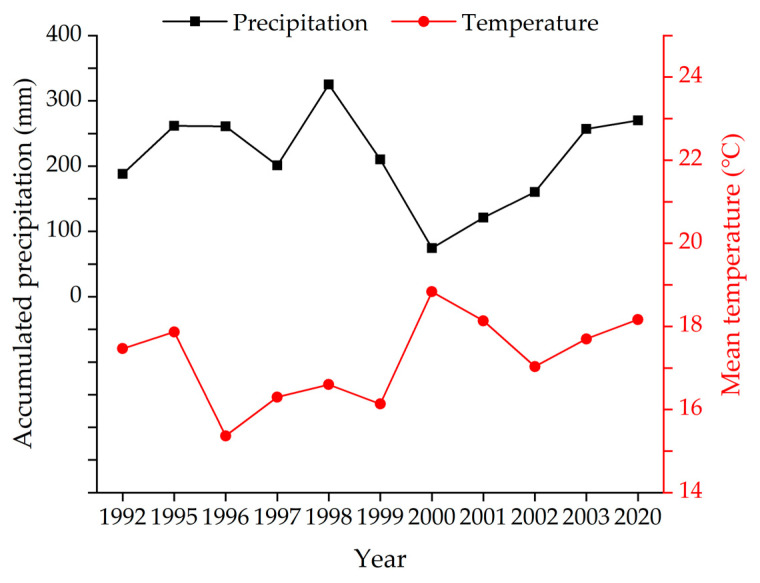
The accumulated precipitation from June to August and the mean temperature from July to September from 1992 to 2003 (except 1993 and 1994) and 2020.

The change of vegetation cover in mining areas depends on natural factors [20,30,31] and artificial factors [32]. In this study area, the natural factors include precipitation, temperature, and topography, and the artificial factors include urban activities and mining activities. The accuracy of the model demonstrates the effectiveness of the selected driving factors. For this study, precipitation, temperature, topography, urban activities, and mining activities were selected as driving factors. Correlation analysis was conducted between FVC and meteorological data (monthly accumulated precipitation and monthly mean temperature, respectively). The Pearson’s correlation coefficients are shown in Table 3. In terms of precipitation, FVC shows the highest correlation with the accumulated precipitation from June to August. In terms of temperature, FVC shows the highest correlation with the mean temperature from July to September. Hence, the accumulated precipitation from June to August and the mean temperature from July to September were used as the driving factors in this study, as shown in Figure 5.

The DEM of the study area was cropped from the ASTER GDEM dataset and used as the driving factor representing topography.

The urban activities and mining activities were quantified with Equations (3) and (4), respectively. There was no mining activity from 1992 to 2003, and mining activities were quantified from 2011 to 2020.

(3) $$x_{urban}=\frac{P_{pop}}{D_{U}+1}$$

where *x_urban_* is the quantified result of urban activities of a pixel, *P_pop_* is the population of Xilinhot, and *D_U_* is the shortest Euclidean distance between the pixel and the urban boundary.

(4) $$x_{mine}=\frac{{MI}_{m}}{D_{M}+1}$$

where *x_mine_* is the quantified result of mining activities of a pixel, *MI_m_* is the coal production, and *D_M_* is the shortest Euclidean distance between the pixel and the mining boundary.

The quantified results of all driving factors were normalized using Equation (5).

(5) $$X_{i}=\frac{x_{i}-x_{min}}{x_{max}-x_{min}}$$

where *X_i_* is the normalized data (i.e., *X_pre_*, *X_temp_*, *X_dem_*, *X_urban_*, and *X_mine_* represent the driving factors for precipitation, temperature, topography, urban activities, and mining activities, respectively), *x_i_* is the quantified data of each driving factor, *x_min_* is the minimum of the quantified data for the driving factor for each year in the study area, and *x_max_* is the maximum of the quantified data for the driving factor for each year in the study area.

**Figure 6:**
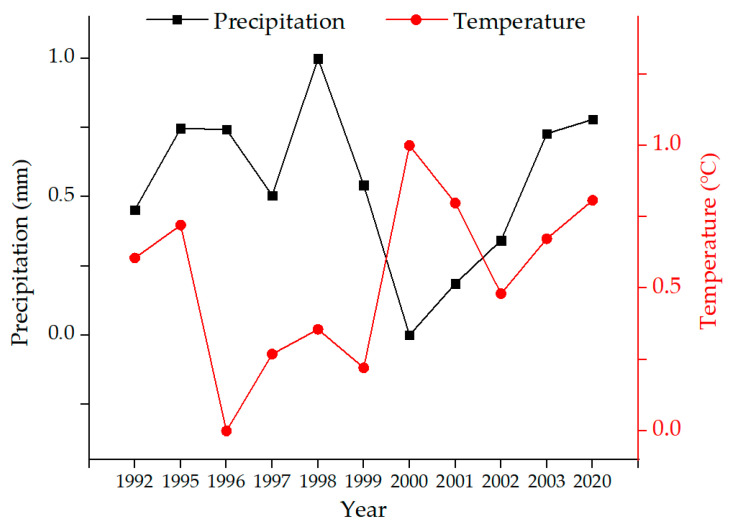
The normalized result of precipitation and temperature from 1992 to 2003 (except 1993 and 1994) and 2020.

**Figure 7:**
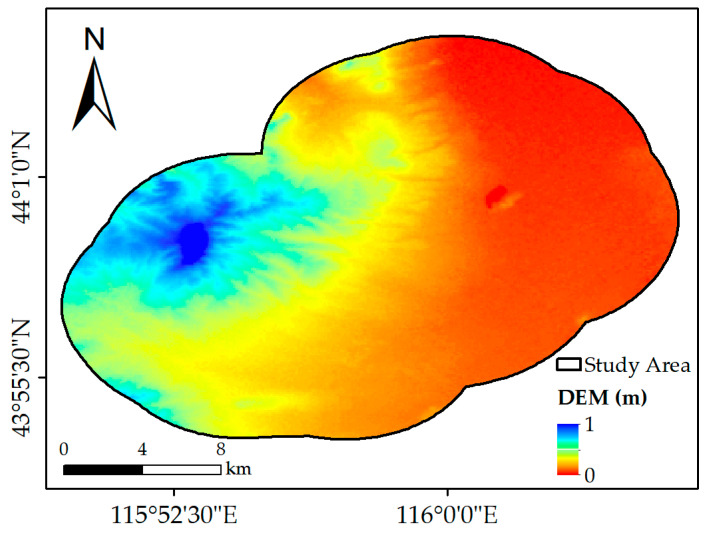
The normalized result of topography.

**Figure 8:**
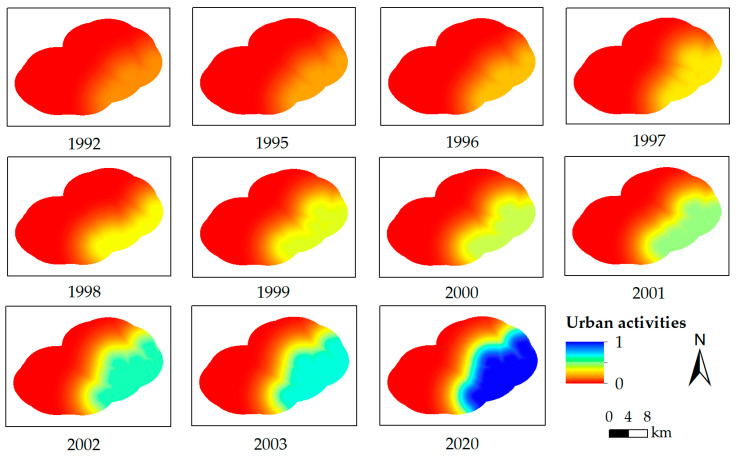
The normalized result of urban activities from 1992 to 2003 (except 1993 and 1994) and 2020.

**Figure 9:**
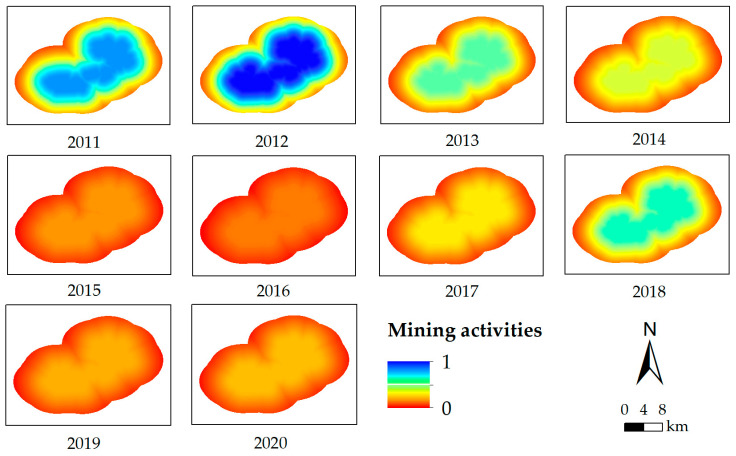
The normalized result of mining activities from 2011 to 2020.

In this study, precipitation and temperature data were used for the Xilinhot station. The topography of the study area did not change significantly during the investigated years, so the same data were used. The normalized results of driving factors in all of the investigated years are shown in Figure 6, Figure 7, Figure 8 and Figure 9.

### 3.3. GWANN

**Figure 10:**
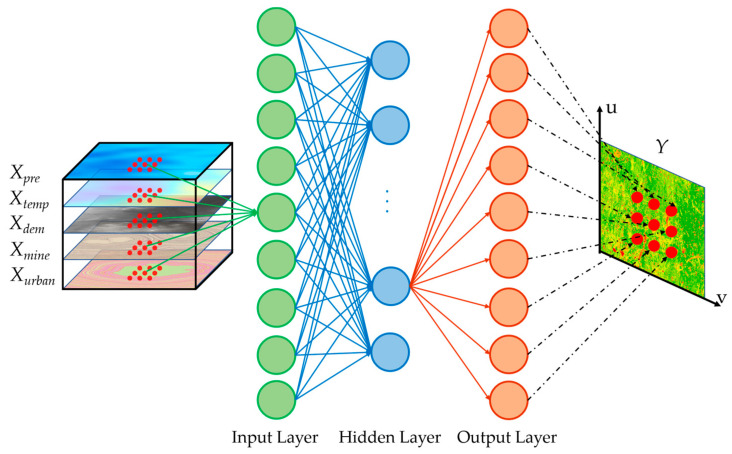
The structure of GWANN. Each neuron in the input layer represents a pixel, and each neuron in the hidden layer is connected to all output neurons.

GWANN is a network for constructing the non-linear and quantitative relationship between the independent variables and the dependent variable [33]. GWANN combines geographical weighting with artificial neural networks. Hence, it can consider the spatial heterogeneity among the driving factors. This network includes the following structure: input layer (driving factors), hidden layer, and output layer (FVC), as shown in Figure 10. The training dataset in this paper includes the pixel location, driving factors (*X_pre_*, *X_temp_*, *X_dem_*, *X_urban_*, and *X_mine_*), and the FVC (*Y*_0_) calculated by the pixel dichotomy model.

The driving factors are passed from the input layer into the hidden layer based on Equations (6) and (7). In neurons, the activation function is a non-linear hyperbolic tangent function, as shown in Equation (8). Each neuron is passed to the neurons at the next layer when Equations (6)–(8) are repeated until reaching the output layer.

(6) $${Layer}_{j}=\underset{i\in S_{j}}{\sum }w_{ij}p_{i}$$

(7) $$p_{i}=\phi {(Layer}_{i})$$

(8) $$\phi \left(x\right)=\frac{e^{x}-e^{-x}}{e^{x}+e^{-x}}$$

where *w_ij_* is the weight of the connection between neuron *i* and neuron *j*, *p_i_* is the output of neuron *I*, *S_j_* is the set of neurons connected to neuron *j*, *Layer_i_* is the input to the neuron *I*, *Layer_j_* is the input to the neuron *j*, *p_i_* is the output of neuron *i*, and *φ*(*x*) is the activation function. 

The error signal is calculated using the backpropagation, as shown in Equation (9) [34]. The error signal depends on the error function, as shown in Equation (10).

(9) $$\delta_{j}=\{\begin{array}{c} \phi^{\prime }\left({Layer}_{j}\right)D_{j}\left(p_{j}-r_{j}\right)\;If\;j\;is\;an\;output\;neuron\\ \phi^{\prime }\left({Layer}_{j}\right)\underset{k}{\sum }\delta_{k}w_{jk}\;else \end{array}$$

(10) $$\;E=\frac{1}{2}\sum_{i=1}^{n}D_{i}{{(r}_{i}-p_{i})}^{2}$$

where *δ_j_* is the error signal of neuron *j*, *p_j_* is the output of neuron *j*, *r_j_* is the *Y*_0_ corresponding to neuron *j*, *w_jk_* is the weight of the connection between neuron *j* and *k*, *δ_k_* is the error signal of neuron k, *E* is the error function, *D_j_* is the geographically weighted distance between the location of the predicted FVC (*Y*) and the output neuron *j*, *φ*′(*x*) is the derivative of the activation function, *r_i_* is the *Y*_0_ corresponding to neuron *i*, *p_i_* is the output of neuron *i*, and *n* is the number of all the pixels (i.e., all the output neurons) in the study area. 

The weights of connection are adjusted using Equation (11).

(11) $$\Delta w_{ij}=-\eta \frac{\partial E}{\partial w_{ij}}=-{\eta \delta }_{j}p_{i}$$

where *η* is the learning rate.

Finally, the value of FVC (*Y*) was predicted by the trained network.

### 3.4. Differential Method

The contribution of the mining activities (denoted as *W_mine_*) on FVC was quantified by the differential method [35].

**Figure 11:**
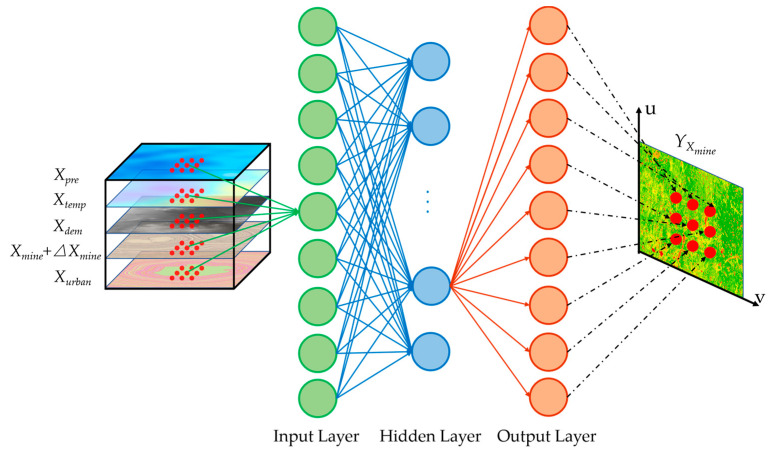
The process of GWANN after adding the bias to a driving factor.

First, the value of a driving factor *X_i_* was multiplied by 0.001 as the bias Δ*X_i_* [35]. When a driving factor was added with a bias, other driving factors remained unchanged. The combination of Δ*X_i_* and the driving factor constructed a new independent variable (*X_i_* + Δ*X_i_*), which formed a set with the remaining independent variables. This set served as the new input layer of the already trained GWANN. Meanwhile, the *Y_X__i_* was obtained by the GWANN. An example of the *X_mine_* is shown in Figure 11.

Second, the partial derivatives of each driving factor were calculated separately, as shown in Equation (12).

(12) $$C_{i}=\frac{Y_{X_{i}}-Y}{\Delta X_{i}}$$

where *C_i_* is the partial derivative of *X_i_* (*C_Pre_*, *C_Temp_*, *C_DEM_*, *C_Urban_*, and *C_Mine_*); Δ*X_i_* is the bias added by *X_i_* (Δ*X_Pre_*, Δ*X_Temp_*, Δ*X_DEM_*, Δ*X_Urban_*, and Δ*X_Mine_*), respectively; *Y_X__i_* is the predicted value after adding bias to *X_i_*; and Y is the predicted value by the original *X_i_*.

Finally, the contribution of mining activities was calculated using Equation (13).

(13) $$W_{\mathrm{mine}}=\frac{C_{\mathrm{mine}}}{\sum_{i=1}^{N}C_{i}}$$

where *W_mine_* is the contribution of the mining activities to FVC and *N* is five (the number of independent variables). Similarly, the contributions of other driving factors to FVC can be obtained (*W_Pre_*, *W_Temp_*, *W_DEM_*, and *W_Urban_*).

### 3.5. Quantifying the Noise in the Contribution of Mining Activities

In principle, a pixel should be identified within the spatial range of mining disturbance if the corresponding contribution of mining activities is more than zero. However, noise is inevitable brought by the differential method.

In the study area, there was no mining activity from 1992 to 2003. In order to quantify the noise in the contribution of mining activities, we assumed the presence of mining activities during this period and calculated the contribution of mining activities, which is a “virtual contribution”. Since there was no mining activity between 1992 and 2003, this “virtual contribution” was taken as the noise in the contribution of mining activities by the differential method.

First, the assumed mining activities were quantified in 1992–2003 (except 1993 and 1994), as shown in Equation (14). Since there was no mining activity from 1992 to 2003, the mining data were provided from 2011 to 2020.

(14) $${\mathrm{vx}}_{\mathrm{mine}}=\frac{{\mathrm{MI}}_{u}}{D_{\mathrm{VM}}+1}$$

where *vx_mine_* is the quantified result of the assumed mining activities, *MI_u_* is the assumed coal production (i.e., the coal production from 2011 to 2020), and *D_VM_* is the shortest Euclidean distance between the pixel and the assumed mining boundary (i.e., the mining boundary from 2011 to 2020).

Second, precipitation, temperature, topography, and urban activities were quantified from 1992 to 2003 (except 1993 and 1994), using the method in Section 3.2. All driving factors were normalized (denoted as *X_pre_*, *X_temp_*, *X_dem_*, *X_urban_*, and *VX_mine_*). In other words, *VX_mine_* is the normalized *vx_mine_*.

**Figure 12:**
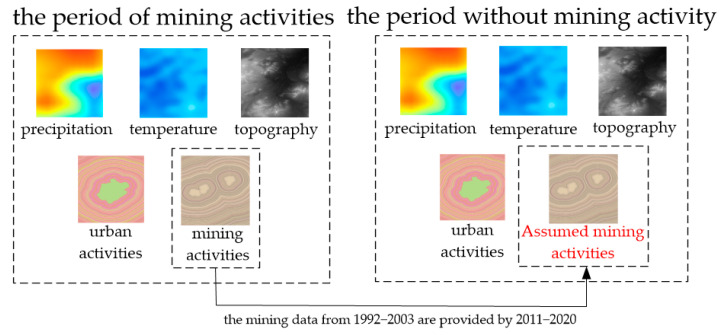
The process of constructing driving factors in the period without mining activity.

Finally, the set of driving factors for the period without mining activity was constructed, as shown in Figure 12.

**Figure 13:**
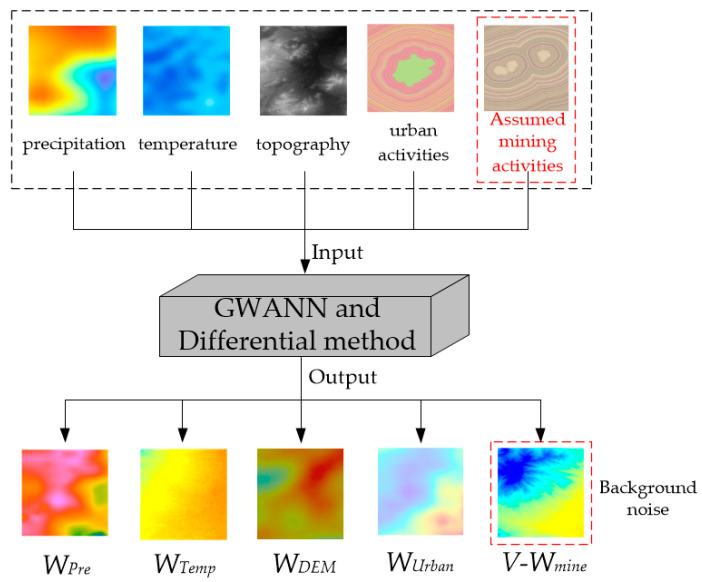
Quantify the virtual contribution of mining activities in the period without mining activity.

Since the contribution could be calculated by simply inputting the quantified data on mining activities in the differential method, the virtual contribution of the mining activities (*V-W_mine_*) could also be calculated from 1992 to 2003 (except 1993 and 1994) using the method in the Section 3.3 and Section 3.4. The process is illustrated in Figure 13.

### 3.6. Significance Test

After repeated experiments and investigations, *V-W_mine_* (i.e., noise in the contribution of mining activities) shows a normal distribution. To judge if the *W_mine_* of a pixel in 2020 belonged to noise or not, a one-sided (right) significance test was applied to the normal distribution of *V-W_mine_* [36]. In this study, when a significance test was performed on the *W_mine_* of a single pixel in 2020, this pixel was called the “tested pixel”. A significance test was used to determine if the tested pixel was significantly disturbed by mining activities as follows. 

In general, statistical significance cannot be claimed if there is more than a 5% chance for the occurrence of the event [37]. In other words, in terms of the principle of a significance test, an event with less than a 5% chance of occurrence is usually referred to as an “unlikely event”, and the null hypothesis should be rejected under this condition, while the alternative hypothesis should be accepted. The significance level α was set to be 0.05 in this study.

The null hypothesis (H_0_) and the alternative hypothesis (H_1_) were listed as follows.

**H_0_:**  *The pixel was not disturbed by mining activities, i.e., W_mine_ ≤ V_0.95_.*

**H_1_:**  *The pixel was disturbed by mining activities, i.e., W_mine_ > V_0.95_.*

**Figure 14:**
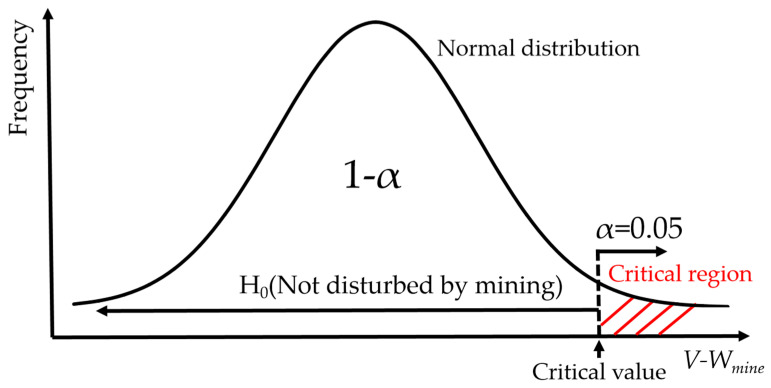
The conceptual illustration of the significance test used in this paper.

*V_0.95_* is the critical value. The critical value (*V_0.95_*) was defined as the value where the cumulative frequency reached 95% on the histogram. When *W_mine_* was in the region where *H*_0_ was rejected (i.e., *H*_1_ was accepted), this region was called the critical region, as shown in Figure 14. If the *W_mine_* of the tested pixel was more than *V_0.95_*, it was identified as the region disturbed by mining activities. Then, all the pixels disturbed by mining activities in the study area were acquired, and these pixels constituted the SRMD.

## 4. Results

### 4.1. Accuracy of GWANN

The root mean square error (RMSE) and mean relative error (MRE) are widely used to evaluate the accuracy of geospatial modeling [38], so these two indicators are used to evaluate the GWANN in this paper, as shown in Equations (15) and (16). The results of the two indicators are shown in Table 4, and the mean *RMSE* for the 11 years of GWANN in the whole study area is 0.0526, while the mean *MRE* is 0.1029.

(15) $$\;\mathrm{RMSE}=\sqrt{\frac{1}{n}\overset{n}{\underset{i=1}{\sum }}{{(\mathrm{FVC}}_{{\mathrm{train}}_{i}}-{\mathrm{FVC}}_{{\mathrm{true}}_{i}})}^{2}}$$

(16) $$\;\mathrm{MRE}=\frac{1}{n}\overset{n}{\underset{i=1}{\sum }}|\frac{{\mathrm{FVC}}_{{\mathrm{train}}_{i}}-{\mathrm{FVC}}_{{\mathrm{true}}_{i}}}{{\mathrm{FVC}}_{{\mathrm{true}}_{i}}}|$$

where *n* is the number of pixels in the study area, *FVC_train__i_* is the *FVC* of pixel *i* predicted by GWANN, and *FVC_true__i_* is the FVC of pixel *i* calculated by the pixel dichotomy model.

### 4.2. The Noise in Contribution of Mining Activities

**Figure 15:**
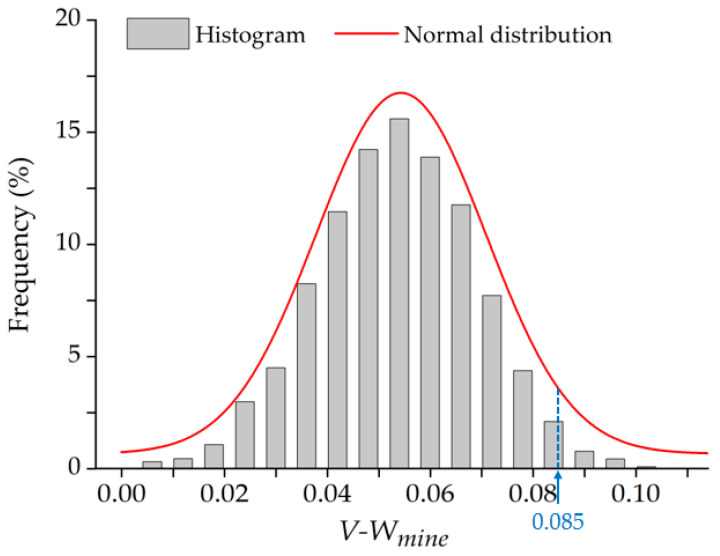
The histogram distribution of the *V-W_mine_*.

The histogram distribution of *V-W_mine_* is shown in Figure 15. The *V-W_mine_* has been fitted to the curve using a Gaussian function, which showed a significant feature of normal distribution. The mean and standard deviation (SD) of *V-W_mine_* were calculated, which were 0.054 and 0.017, respectively. By calculating the cumulative frequency, the critical value (*V_0.95_*) was found to be 0.085 (Figure 15).

### 4.3. Results of the Identified SRMD

**Figure 16:**
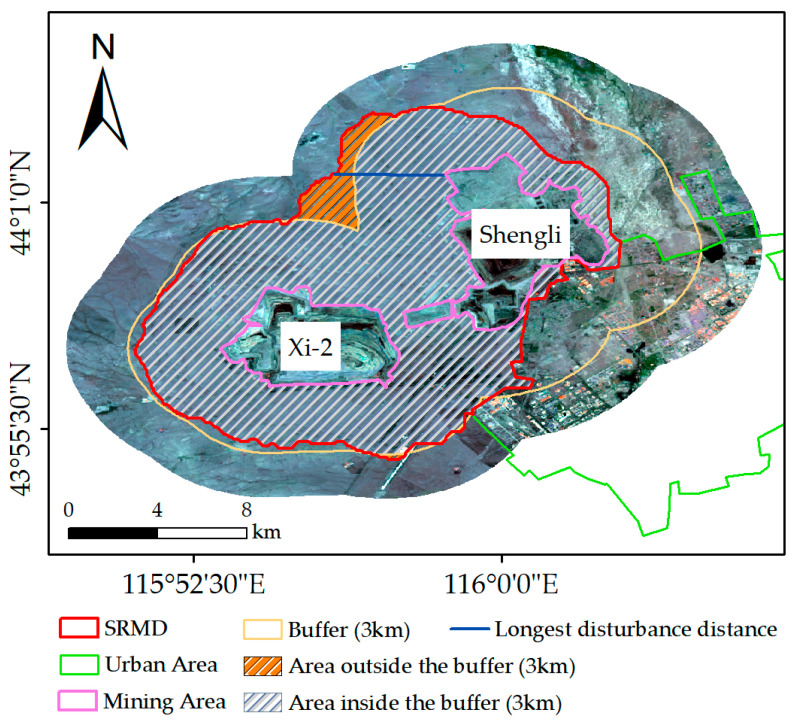
The identified SRMD in 2020.

**Figure 17:**
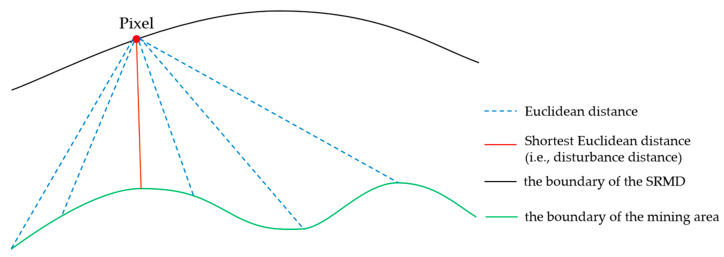
The conceptual illustration of the disturbance distance.

**Figure 18:**
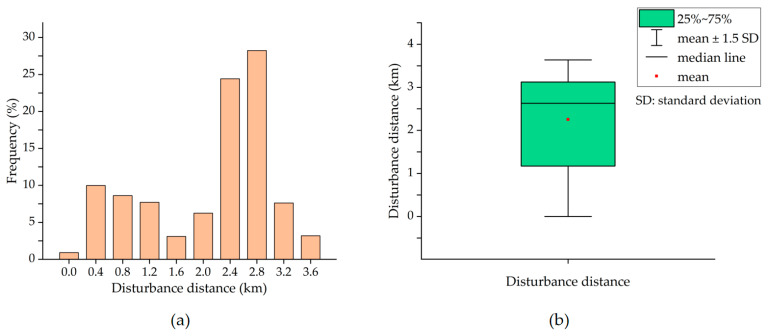
The statistics of the disturbance distance. (**a**) The histogram distribution of the disturbance distance; (**b**) the box-line diagram of the disturbance distance.

The pixels disturbed by mining activities were selected by a significance test, and the identified SRMD in 2020 is shown in Figure 16. Distance measurements were performed on the map displayed in Figure 16. The shortest Euclidean distance between the pixel on the boundary of SRMD and the boundary of the mining area was calculated and defined as the “disturbance distance”. The conceptual illustration of the disturbance distance is shown in Figure 17. The statistics of the disturbance distance are shown in Figure 18.

A buffer of 3 km beyond the boundary of each mining area in 2020 was generated (Figure 16). Figure 16 suggests that most of the SRMD are inside the 3 km buffer from the boundary of the mining area. The average and median disturbance distance is 2.25 km and 2.63 km, respectively, while the middle of the distance values is distributed in 1.17 km~3.12 km (i.e., 25~75%) (Figure 18). The area of the SRMD that exceeds the 3 km buffer is the area adjacent to both the Xi-2 Mine and Shengli Mine (Figure 16). In this area, the longest disturbance distance in the whole SRMD was present, which was 3.63 km (Figure 13). The area of the SRMD that did not exceed the 3 km buffer was mainly located to the southeast and northeast of the Shengli Mine (Figure 16). The shortest distance was 0 km (Figure 16), located in the area where the Shengli Mine borders the urban area.

**Figure 19:**
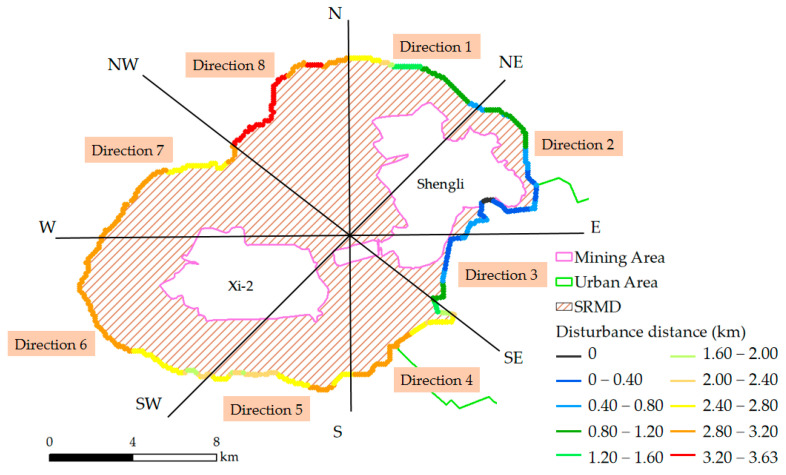
The disturbance distance in different directions.

The disturbance distance in different directions is shown in Figure 19. The SRMD was divided into eight regions according to different directions (directions 1–8, Figure 19). The disturbance distance in direction 8 was between 2.8 km and 3.63 km, which was significantly higher than that in other directions. The disturbance distance in direction 2 and direction 3 were between 0 and 1.2 km, which was significantly lower than that in the other directions. In addition, the disturbance distance in direction 1 was between 0.4 km and 2.8 km. The disturbance distance in other directions (4–7) was between 1.6 km and 3.2 km.

## 5. Discussion

In this study, the coupling of multiple factors was considered, and the mining factor was separated from other factors to identify the SRMD by significance test. Since the contribution of the mining factor was quantified, the separate impact of mining activities on the environment can be understood more clearly and visually. Meanwhile, some results need further explanation and discussion.

The GWANN was used to construct the relationship between FVC and five driving factors, and the differential method was used to quantify the contribution of mining activities to FVC. The mean RMSE and MRE for the 11 years were used to evaluate the accuracy, which was 0.0526 and 0.1029 in the study area, respectively. The low values of the two error indicators suggest that GWANN and the differential method have been successfully applied to quantify the contribution of mining activities in this study.

Figure 15 demonstrates that the histogram of *V-W_mine_* obeys normal distribution. In particular, the *V-W_mine_* is between 0 and 0.085 with a 95% probability. In other words, there is a very low virtual contribution of mining activities and its normal distribution during the period without mining activities. These results are highly consistent with our assumption that the calculated virtual contribution (*V-W_mine_*) is the noise in the contribution of mining activities brought by the differential method. What’s more, this high consistency with our assumption suggests the feasibility and effectiveness of the proposed method for identifying the SRMD based on contribution quantification and significance test.

Most of the SRMD are distributed in the buffer of 3 km (Figure 16) with an average disturbance distance of 2.25 km (Figure 18). The results in Figure 16 and Figure 18 have described the characteristics of the spatial range of mining disturbance on FVC. In particular, the longest disturbance distance is present in the area adjacent to both the Xi-2 Mine and Shengli Mine, which indicates that the superimposed impact from the two mines led to the larger spatial range of mining disturbance. The superimposed disturbance may be the reason why the high values of disturbance are present in direction 8 (Figure 19). However, in direction 4, also adjacent to both two mines, the distance is significantly less than that in direction 8 (Figure 19). In fact, the distance in directions 2–4 is generally shorter than that in other directions; even the shortest disturbance is present in direction 2. Moreover, the urban area is adjacent to the mining area in directions 2–4. Hence, it can be inferred that the disturbances in directions 2–4 are mainly from the urban activities and the mining disturbance is not significant. Although the regions in directions 2–4 are adjacent to the urban area, the disturbance distance is higher in direction 4 than that in the other two directions (Figure 19), which also indicates the superimposed impact from the two mines. In direction 1, there are river valleys and woodlands where the quality of the background environment is relatively higher than that in other areas, which may be the reason why a short disturbance distance is present in this direction. Figure 19 demonstrates that the spatial range of mining disturbance in the study area possessed significant directional heterogeneity. Overall, the results in this paper provide important data for understanding the indirect impact of mining activities on the surrounding environment in the study area and are useful for the plan of environmental rehabilitation.

This paper makes up for some deficiencies in existing publications. Here, several advantages of this study are discussed as follows. 

(1) Elimination of coupling impact. Compared to direct observations of vegetation indices (e.g., [9,10,11,12,13,14]), the quantified contribution of the mining factor eliminates the impact of other factors. The vegetation in the study area is impacted by the coupling of multiple factors, so it is more rational to obtain the results of SRMD in 2020 with this study method.

(2) Spatial continuity. Sample areas selected from different directions or buffers generated at different distances were investigated by some methods (e.g., [10,13]), and the spatial discontinuity between sample areas creates blind areas in the study. In contrast, the contributions of the driving factors to FVC of this study method are calculated at the pixel scale. In other words, the contribution of each driving factor is spatially continuous. Hence, the SRMD obtained is also spatially continuous.

(3) Threshold stability. It is more scientific and effective to calculate the critical value by significance test than to determine the threshold artificially (e.g., [8,9,10,11,12]). Moreover, the significance test avoids the instability brought by the artificial determination of a threshold.

However, we recognize that there are still some shortcomings that should be improved in the future. The SRMD in 2020 is identified in this paper. Subsequently, the change of SRMD in long time series can be investigated, and the pattern in temporal changing is possible to be discovered. In terms of data, five driving factors (precipitation, temperature, topography, urban activities, and mining activities) are selected to construct GWANN in this paper, and more driving factors can be considered for analysis in subsequent investigations. There are different driving factors in different conditions of natural geography, and the appropriate driving factors should be selected. The method of this study may be applied when the appropriate driving factors are selected under different conditions of natural geography. The proposed method is subject to further verification of adaptation in other mining areas.

## 6. Conclusions

In this study, GWANN was used to construct a non-linear and quantitative relationship between FVC and five driving factors. The differential method was used to quantify the contribution of mining activities to FVC. The noise in the contribution of mining activities (i.e., the virtual contribution *V-W_mine_*) was calculated during the period without mining activities (1992–2003, except 1993 and 1994). The SRMD in 2020 was identified utilizing the significance test. Some conclusions were reached as follows.

(1) The accuracy of GWANN demonstrates the effectiveness of GWANN and that the differential method can be used to quantify the contribution of mining activities in this study area.

(2) The noise in the contribution of mining activities to FVC obeys normal distribution, and the value of noise was relatively low. The normal distribution of noise with low values demonstrates the usability of the significance test for judging if a region is disturbed by mining activities.

(3) In the study area, most of the SRMD are inside the 3 km buffer with an average disturbance distance of 2.25 km for the whole SRMD. The longest disturbance distance is present in the area of superimposed impact from the two mines, and the shortest disturbance distance is in the area adjacent to the urban. Significant directional heterogeneity is possessed by the SRMD.

In this study, the SRMD in 2020 was identified with the way of unmixing the coupling impact, spatial continuity, and determining threshold by significance test (not artificial determination). The pattern of SRMD in long time series and more driving factors should be investigated in the future.

## Figures and Tables

**Table 1:** List of band information for Landsat images.

Band Name	Landsat 5 TM		Landsat 7 ETM+		Landsat 8 OLI	
	Band Number	Bandwidth (μm)	Band Number	Bandwidth (μm)	Band Number	Bandwidth (μm)
Coastal	--	--	--	--	Band 1	0.43–0.45
Blue	Band 1	0.45–0.52	Band 1	0.45–0.52	Band 2	0.45–0.51
Green	Band 2	0.52–0.60	Band 2	0.52–0.60	Band 3	0.53–0.59
Red	Band 3	0.63–0.69	Band 3	0.63–0.69	Band 4	0.64–0.67
NIR	Band 4	0.76–0.90	Band 4	0.77–0.90	Band 5	0.85–0.88
SWIR-1	Band 5	1.55–1.75	Band 5	1.55–1.75	Band 6	1.57–1.65
TIR	Band 6	10.40–12.50	Band 6	10.40–12.50	--	
SWIR-2	Band 7	2.08–2.35	Band 7	2.09–2.35	Band 7	2.11–2.29
Pan	--	--	Band 8	0.52–0.90	Band 8	0.50–0.68
Cirrus	--	--	--	--	Band 9	1.36–1.38

**Table 2:** Information for each dataset.

Data	Data Sources	Resolution
Landsat images	Google Earth Engine (GEE)	30 m
Precipitation	The website of China Meteorological Data (http://data.cma.cn (accessed on 2 February 2021))	Station
Temperature	The website of China Meteorological Data (http://data.cma.cn (accessed on 2 February 2021))	Station
Topography	ASTER GDEM	30 m
Urban activities	Administrative boundary and Xilinhot statistical yearbook	At the scale of town and village in Xilinhot and the total population of each year
Mining activities	Mining companies	Annual coal production at each mine

**Table 3:** The Pearson’s correlation coefficients of FVC with the accumulated precipitation and the mean temperature.

Precipitation (mm)	Month	Pearson’s correlation coefficient	Temperature (°C)	Month	Pearson’s correlation coefficient
	January	0.267		January	0.276
	February	0.125		February	−0.023
	March	0.047		March	0.475
	April	−0.020		April	0.088
	May	−0.156		May	−0.224
	June	0.319		June	−0.165
	July	0.625 *		July	−0.407
	August	0.255		August	−0.308
	September	−0.018		September	−0.354
	October	−0.361		October	0.097
	November	0.110		November	0.402
	December	0.110		December	0.402
	June–August	0.660 **		June–August	−0.500
	July–September	0.497		July–September	−0.616 *
	July–August	0.554 *		July–August	−0.533 *

* At the 0.05 level; ** at the 0.01 level. Significant correlation.

**Table 4:** Accuracy of the GWANN.

Year	1992	1995	1996	1997	1998	1999
RMSE	0.0117	0.0191	0.0468	0.0043	0.0261	0.0422
MRE	0.0254	0.0420	0.1193	0.0105	0.0554	0.1026
Year	2000	2001	2002	2003	2020	
RMSE	0.0261	0.0806	0.0658	0.1015	0.1546	
MRE	0.0561	0.1521	0.1275	0.1895	0.2518	

## Data Availability

The Landsat data and topography data can be downloaded from GEE (https://developers.google.com/earth-engine/datasets/, accessed on 2 February 2021), and the precipitation and temperature data can be downloaded from the China Meteorological Data Network (http://data.cma.cn, accessed on 2 February 2021).
